# Influence of Prosocial Motivation on Employee Creativity: The Moderating Role of Regulatory Focus and the Mediating Role of Knowledge Sharing

**DOI:** 10.3389/fpsyg.2021.704630

**Published:** 2021-09-14

**Authors:** Xizhou Tian, Xiqiang Peng, Xiaoping Peng

**Affiliations:** ^1^School of Business Administration, Chongqing Technology and Business University, Chongqing, China; ^2^Business School, Nankai University, Tianjin, China

**Keywords:** prosocial motivation, employee creativity, knowledge sharing, promotion focus, prevention focus

## Abstract

Stimulating and improving the creativity of employees are both theoretically and practically important. The relationship between prosocial motivation and creativity has gradually gained attention in recent years; however, in the context of controlling for intrinsic motivation, the influence process and results between the two are not yet clear. Based on the motivated information processing model, componential theory of creativity, and regulatory focus theory, this study analyzed the mediating role of knowledge sharing and the moderating role of regulatory focus in the relationship between prosocial motivation and the creativity of employees. For this, we used the PROCESS program and the bootstrap method to test the theoretical hypotheses. Consequently, a survey of 320 Chinese employees revealed that, under the condition of controlling for intrinsic motivation, the prosocial motivation of employees was positively related to creativity and partially mediated by knowledge sharing. Furthermore, regulatory focus negatively moderated the correlation between prosocial motivation and knowledge sharing. Specifically, we found that the higher the prevention focus was, the weaker the effect prosocial motivation had on knowledge sharing. Contrary to the hypothesis, promotion focus also played a negative moderating role. Thus, the results revealed the mechanism and boundary conditions of prosocial motivation on creativity. This study expands the research on prosocial motivation and provides guidance on how managers can enhance the creativity of their employees.

## Introduction

In the context of the increasingly fierce global market competition of today, the innovation ability of an enterprise is related to its survival and development. In particular, one of the important sources for improving innovation ability is the creativity of organization members (Zhou and Hoever, 2014). Therefore, effectively stimulating and releasing the creativity of employees is particularly important and urgent. Motivation has long been a psychological process that stimulates, guides, and maintains human behavior (Gilmore, 2013). Understanding the motivational basis of creativity is one of the long-term goals of creativity research (Amabile and Pillemer, 2012). Among these goals, prosocial motivation, which is defined as the willingness to work hard for the well-being of others, has attracted much attention in the field of creativity research because it helps employees go beyond the limitations of their own perspectives and enhance individual empathy and creative thinking (Grant and Berry, 2011; Li and Bai, 2015a; Liu et al., 2016). In particular, the theme of prosocial motivation as a “social glue” for the harmonious coexistence of different classes and ethnicities has more contemporary significance in the context of indifferent interpersonal relationships.

The relationship between prosocial motivation and creativity has gradually attracted the attention of scholars (De Dreu et al., 2011; Grant and Berg, 2011; Grant and Berry, 2011; Li and Bai, 2015a); however, the results are not sufficient. On the one hand, when current scholars discuss the relationship between prosocial motivation and employee creativity, they often regard prosocial motivation as a boundary condition in the relationship between intrinsic motivation and creativity (Bechtoldt et al., 2010; De Dreu et al., 2011; Grant and Berry, 2011). Although some studies have verified the influence of prosocial motivation on creativity, the role of intrinsic motivation has been ignored (Li and Bai, 2015b; Zhang and Liu, 2018). In fact, prosocial motivation still plays an important role in creativity after controlling for intrinsic motivation (Liu et al., 2016). Therefore, it is necessary to further clarify the relationship between prosocial motivation and creativity from an empirical perspective under the condition of controlling for intrinsic motivation. On the other hand, there is no consensus on the relationship between prosocial motivation and employee creativity. Grant and Berry (2011) and Li and Bai (2015a,b) confirmed a positive correlation between prosocial motivation and creativity. In contrast, an experiment performed by Boeck (2015) on young people showed that prosocial motivation had no positive effect on creativity. However, few empirical studies have explored and analyzed the aforementioned inconsistent conclusions. Studies have also confirmed that there are cultural differences in creativity performance (Morris and Leung, 2010). At present, most research objects are from Western culture, whereas, in the typical collectivist culture of China, the prosocial behaviors of helping and considering the interests of others have always been encouraged (Hofstede, 2001; Li et al., 2012). Thus, the relationship between prosocial motivation and creativity and its mechanism of action are worthy of further discussion.

According to the componential theory of creativity, domain knowledge is an indispensable element of creativity (Amabile, 1996). Specifically, knowledge sharing is crucial for employees to exchange knowledge and jointly create new knowledge (Van den Hooff and De Ridder, 2004). As a kind of helping behavior, the occurrence of knowledge sharing is mainly affected by internal motivation (Lombardi et al., 2020). Whether an individual is a juvenile or an employee, their prosocial motivation may prompt them to show more knowledge sharing behaviors (Asterhan and Bouton, 2017; Jin et al., 2020). In fact, employees are not only knowledge sharers, but also potential beneficiaries when they share knowledge. To effectively share knowledge, sharers not only generate new ideas about existing knowledge by reorganizing it but can also deepen their understanding of shallow knowledge in the process of communicating with others, all of which provide knowledge for the generation of creativity reserves (Bhatti et al., 2020). In recent years, studies have deeply explored the mediating effects of knowledge sharing on different outcomes based on different theoretical perspectives. In particular, studies have found that knowledge sharing is an important path in the formation of employee creativity and organizational innovation (Zhu and Chen, 2013; Bhatti et al., 2020), which provides a theoretical basis for this study to introduce knowledge sharing as a mediating variable to deeply explore the “black box” of prosocial motivation and creativity.

The process of prosocial motivation-stimulating behavior is essentially a process of behavior regulation, which is affected by individual regulatory focus (Chen et al., 2016). The so-called regulatory focus includes two dimensions, namely, promotion focus and prevention focus, where the former emphasizes success, while the latter focuses on risk aversion (Higgins, 1989). The regulatory fit theory suggests that, when individuals complete tasks in ways or behaviors that are consistent with their regulatory focus orientation, this kind of internal fitting enables them to experience the joy of the tasks more. The consistency of mood and behavior also promotes their recognition of the behavior. Thus, they are more willing to show behavior in line with their focus orientation (Higgins, 2000a). Meanwhile, the active tendency of prosocial motivation also has a different degree of compatibility with the approach and avoidance strategies of the regulatory focus. Studies have shown that regulatory focus not only affects knowledge sharing behavior (Shin et al., 2016), but also affects individual information processing and behavioral orientation (Werth and Foerster, 2007), which, in turn, lead to differences in individual behavior under the influence of behavioral motivation. For example, compared with employees with a high prevention focus, it has been shown that higher prosocial motivations of employees with low prevention focus lead them to exhibit more accelerative voice behaviors (Chen et al., 2016). Furthermore, in the context of time constraints, employees with high regulatory focus are more likely to share knowledge to achieve team goals (Ju et al., 2019). Overall, because knowledge sharing is proactive and difficult to enforce, a subconscious recognition can better motivate an employee to share their knowledge with others. The above analysis provides the theoretical enlightenment for this study to bring the individual regulatory focus into the research framework and explore the moderating role of regulatory focus between prosocial motivation and knowledge sharing.

In summary, to answer the question on how prosocial motivation affects creativity, the purpose of this research was to rely on the motivational information processing theory to explore the mechanism and boundary conditions of the aforementioned relationship. Specifically, under the condition of controlling for intrinsic motivation, this study verified the influence of the prosocial motivation of employees on creativity, the mediating role of knowledge sharing between these two variables, and the moderating role of regulatory focus in the impact of prosocial motivation on knowledge sharing. Theoretically, this study revealed the unique role of prosocial motivation in creativity and its influencing processes, which bridges the gap in existing research. In practice, the discussion of these questions can help managers improve employee creativity in a targeted manner.

## Theoretical Framework and Hypotheses

### Prosocial Motivation and Creativity

According to the motivated information processing model, social motivation affects the content and direction of information processing. The desire of the employees also determines, to some extent, how they process information (Nijstad and De Dreu, 2012). Prosocial motivation, in particular, describes the desire to benefit others or expend effort out of concern for others (Grant, 2008). It helps employees go beyond the limitations of their own perspectives, improve their sensitivity to the views and needs of others, and perform tasks to the best of their abilities (De Dreu et al., 2011), all of which are crucial for generating creativity (Li and Bai, 2015a,b). Specifically, Zhang and Bartol (2010) suggested that the degree of individual participation in the creative process depends on the degree of concern regarding the problem. Prosocial employees who are driven by the greater interests of others, the organization, or groups are more concerned about the well-being and needs of others (De Dreu, 2006). Therefore, they are more involved in creative work (Le, 2015) and often invest more time, energy, and resources in their work to absorb and master domain and creative skills and form more flexible cognitive structures and in-depth strategies to deal with challenging problems, thereby enhancing creativity. It is also because of the concern for the well-being of others that employees with prosocial motivation are able to better perceive the value of their work for others or for the organization. This helps to enhance their sense of mission and self-identity, makes them more inclined to seek multiple solutions to solve problems (Ma and Zhao, 2015), and helps them show greater perseverance and persistence in the process of solving a problem (Grant, 2008). At the same time, prosocial motivation drives employees to consider problems and obtain information from the perspective of others, which means that they will filter useless information when thinking about solutions and generate useful ideas that are not only novel, but also suitable for solving the problems or meeting the needs of others (Grant and Berry, 2011). In addition, some scholars have pointed out that individuals with high prosocial motivation usually show more compassion and generosity to generate more positive emotions (Hoever et al., 2012; Carmeli et al., 2014), which consequently help to increase creativity. This shows that employees driven by prosocial motivation expect their work to contribute to the well-being of others; therefore, they are able to think from the perspective of others, enhance the degree of individual participation and the ability to integrate viewpoints, and thus promote the enhancement of creativity. Given that the study of prosocial motivation is still in its infancy, current studies have initially confirmed the positive impact of intrinsic and prosocial motivations on creativity (Grant and Berry, 2011; Li and Bai, 2015a). However, a meta-analysis found that prosocial motivation has a unique contribution to creativity, and controlling for intrinsic motivation is more conducive to investigating the exclusive role of prosocial motivation (Liu et al., 2016). Based on the above analysis, we proposed the following hypothesis:

**Hypothesis 1**. Under the condition of controlling for intrinsic motivation, prosocial motivation is positively related to the creativity of employees.

### The Mediating Role of Knowledge Sharing

While some studies have shown that prosocial motivation has a positive impact on employee creativity, scholars are also exploring the underlying mechanisms of the aforementioned relationship (Li and Bai, 2015b; Pan and Bai, 2017). Today, in the knowledge society, knowledge sharing is a process whereby people exchange knowledge and create new knowledge (Van den Hooff and De Ridder, 2004); it is also a process of continuous interaction and generosity. It focuses on providing information to facilitate problem-solving, creativity, innovation, or change (Wang and Noe, 2010). In this study, we proposed that prosocial motivation facilitates knowledge sharing, which, in turn, nurtures employee creativity.

On the one hand, based on the motivated information processing theory, the desires of individuals can shape the way they react to information (De Dreu, 2006). In particular, individuals with prosocial motivation are more likely to connect the experiences of others with their own and empathize with others, show concern for others, and identify with the experiences of others (Aron et al., 1991; Sun et al., 2020a). Therefore, employees with higher levels of prosocial motivation may prioritize the needs of their coworkers (Grant, 2008) and be more inclined to respond favorably to requests for assistance. Empirical studies have shown that prosocial motivation is correlated with help-giving (Rioux and Penner, 2001) and help-seeking behaviors, employees experiencing the meaningfulness of their work, and resource and information sharing (Utz et al., 2014). In organizational knowledge management, knowledge contributors with higher prosocial motivation exhibit a high level of knowledge sharing and, consequently, a low level of knowledge hiding (Škerlavaj et al., 2018). In addition, employees driven by prosocial motivation often strive to build good relationships with their colleagues and develop positive perceptions and attitudes to create a harmonious working environment for themselves (Bolino et al., 2012). In collectivist societies such as China, individuals are particularly concerned about the interrelationships among people (Takeuchi et al., 2015). Therefore, when other members of the organization need help, prosocial individuals actively express their ideas and opinions because of their willingness to help others, thus promoting the transmission of information and knowledge. Knowledge sharing is an extra-role behavior that is conducive to the development of organizations (Srivastava et al., 2006), which focuses on providing information to facilitate problem solving or change (Wang and Noe, 2010). It is also, therefore, regarded as a prosocial behavior (Bolino and Grant, 2016). Thus, it can be inferred that the prosocial motivation of an individual has a significant positive impact on knowledge sharing. In fact, some studies have confirmed that prosocial motivation can promote organizational citizenship behavior (Takeuchi et al., 2015) and a series of prosocial behaviors in the Chinese context, such as helping and giving advice (Chen et al., 2016; Lu et al., 2017).

On the other hand, the componential theory of creativity proposes that individual knowledge is conducive to the generation of creativity (Amabile, 1996). Thus, employees who share their knowledge can enhance their own creative thinking and knowledge, thereby enhancing their own creativity. In terms of the sharing process, individuals must reorganize their knowledge so that they can transfer knowledge accurately. This knowledge may have been neglected before; however, once it is extracted from the mind and related to other information, the sharing process itself may become a source of inspiration (Zhong et al., 2015). From the social exchange and learning perspectives, knowledge recipients are more willing to pass on knowledge and information to the sharers based on the principle of reciprocity (Jin, 2013), which means that sharers can also learn from the experience and techniques of others. In addition, by assisting others in solving problems, employees can acquire new insights and skills themselves (Shah et al., 2015). The basic knowledge and professional skills of employees are one of the components of creativity (Amabile, 1997) that are conducive to the creation of new ideas. Moreover, several studies have also proven that knowledge sharing is positively related to employee creativity (Khazanchi and Masterson, 2011; Zhang et al., 2016).

According to the above analysis, based on the motivated information processing theory, employees with prosocial motivation expect their work to contribute to the well-being of others and are often more willing to display helpful behaviors, such as knowledge sharing, out of kindness toward others and responsibility to the organization. According to the componential theory of creativity, employees driven by prosocial motivation can exchange ideas and process information in the sharing process; thus, knowledge sharers can deepen not only their understanding of existing knowledge but also update their own knowledge and skills, which is a prerequisite for creative ideas. Based on this, we can assume that prosocial motivation affects creativity through the mediating role of knowledge sharing. Therefore, we proposed the following hypothesis:

**Hypothesis 2**. Knowledge sharing mediates the positive relationship between prosocial motivation and the creativity of employees.

### The Moderating Role of Regulatory Focus

Employees driven by prosocial motivation often exhibit organizational citizenship behaviors, such as knowledge sharing (Škerlavaj et al., 2018). However, the process of prosocial motivation in stimulating behavior is, essentially, a process of behavioral regulation, which is affected by individual regulatory focus (Chen et al., 2016). The regulatory focus theory proposes a self-regulation process in which individuals associate themselves with goals (Higgins, 1997). This process consists of two dimensions: promotion focus and prevention focus. Promotion focus is a self-regulation tendency related to growth needs (e.g., ambitions, ideals, desires, etc.). Specifically, individuals with promotion focus are sensitive to positive results and more often adopt approach strategies to pursue their own goals. Research has found that individuals with high promotion focus are more concerned about hope and achievement, sensitive to rewards and losses, and have a higher risk appetite. Such individuals often show stronger internal driving forces in their work, are open and optimistic in the face of problems, strive to pursue breakthroughs in the status quo instead of sticking to conventions, and are more willing to exceed their duties (Friedman and Förster, 2001; Luan and Zhang, 2020). On the contrary, prevention focus is a self-regulation tendency related to safety requirements (e.g., self-protection, protection from harm, etc.). In particular, individuals with high prevention focus are more concerned about duties and obligations, are more sensitive to the occurrence and absence of punishment, and have lower risk appetites. They also tend to adopt avoidance strategies to achieve their goals in their work and focus on preventing mistakes and losses. Compared with individuals with a high promotion focus, these individuals exhibit more vigilant behaviors at work, are less optimistic when facing problems, and tend to avoid challenges and maintain the status quo (Crowe and Higgins, 1997; Brockner and Higgins, 2001; Friedman and Förster, 2001). Furthermore, individuals usually adopt behaviors that fit their own orientation types and levels (Lanaj et al., 2012; Cao and Xu, 2017; Mao, 2017). Therefore, when people are faced with the difficult choice of sharing their knowledge, the individual regulatory focus will lead to differences in the actual behaviors of individuals under the influence of behavioral motivations (Ju et al., 2019). Based on the above discussion, we believe that regulatory focus may moderate the relationship between prosocial motivation and knowledge sharing.

Knowledge is an important resource for employees to stand out from their peers. Employees who share their knowledge are likely to feel worthy and gain the favor of others (Lin, 2007). However, sharing knowledge also means the loss of knowledge ownership, which may threaten the status and power of an individual in an organization (Wang et al., 2020). Therefore, knowledge sharing is a behavior in which both risks and opportunities coexist (Huo et al., 2016; Park et al., 2017). For instance, promotion-focused individuals may pursue their ideals and personal values, or they may be eager to achieve team goals; therefore, they are more inclined to engage in more knowledge-sharing behaviors (Li et al., 2013; Shang et al., 2016). With this, individuals can also strengthen the relationship between their motivation and knowledge-sharing behavior due to the fitting effect of their own regulatory focus and internal motivation. On the one hand, when behaviors guided by prosocial motivation are consistent with the preference of the employees for promotion focus, prosocial motivation promotes the formation of the regulatory fitting. Specifically, individuals with prosocial motivation break through the principle of self-centered interests and often think about problems from the perspective of others, consequently showing more extra-role behaviors (Zhu and Chen, 2013). Furthermore, individuals with a high promotion focus often use approach strategies to achieve their goals; thus, they are more likely to receive support from others and show higher returns and helpful behaviors (Gorman et al., 2012; Lanaj et al., 2012), which fits the extra-role behavior guided by prosocial motivation. According to regulatory fit theory, this consistent adjustment and the sense of fitting these employees have enabled them to further strengthen their cognition or motivation for sharing (Higgins, 2000b; Lei et al., 2015). On the other hand, the other orientation of prosocial motivation makes employees pay more attention to the well-being of others and the collective, thus making them regard themselves and the organization as a community of interests (Sun et al., 2020b). To match this, employees with high promotion focus have a higher emotional commitment and form strong emotional attachments and identification with the organization (Gorman et al., 2012). Therefore, this fitting may strengthen the prosocial motivation of these employees to pay attention to the common interests of the organization. This, in turn, stimulates the willingness of employees to contribute to the organization, thus showing more knowledge sharing and other behaviors that are beneficial to the organization (Zhou et al., 2016). Therefore, we proposed the following hypothesis:

**Hypothesis 3**. Promotion focus positively moderates the relationship between prosocial motivation and knowledge sharing. Compared with employees with a low promotion focus, employees with prosocial motivation and high promotion focus are more likely to show knowledge sharing.

Individuals with a high prevention focus pay more attention to negative results, strive to avoid risks, and adopt avoidance strategies to achieve their goals (Mao, 2017). To them, the process of knowledge sharing is not only accompanied by a series of time and emotional costs, but may also lead to the loss of the dominant position of the sharer in an organization (Chen et al., 2018). Such a “dangerous” signal is easily detected by prevention-focused individuals. Therefore, they are more inclined to regard knowledge sharing as an uncontrollable risk. To avoid potential losses, they subsequently generate conservative response mechanisms, which may result in greater knowledge hiding (Shang et al., 2016). Therefore, individuals with high prevention focus, who are afraid of losing to their competition, are not very willing to share their knowledge and skills with others. It has also been proven that such employees are less likely to support others or receive support from others (Gorman et al., 2012; Lanaj et al., 2012; Righetti and Kumashiro, 2012). These characteristics are mutually exclusive with the characteristics of organizational citizenship behavior, such as helping others, driven by prosocial motivation. In addition, individuals with a high prevention focus are more likely to fulfill their tasks and responsibilities in accordance with the system rules and set the lowest performance standards for themselves (Cao and Xu, 2017). However, knowledge sharing is an extra-role behavior beyond the minimum job requirements (Peng et al., 2019). This mismatch between behavioral patterns and regulatory orientation further weakens previous cognition and motivation (Higgins, 2000a), also consequently weakening the promoting effect of prosocial motivation on knowledge sharing. In addition, from the perspective of emotion and cognition, individuals with high prevention focus are afraid of failure and adopt avoidance strategies to achieve their goals more often. However, prosocial motivation also makes individuals willing to help others. This contradiction makes it difficult for these individuals to choose between helping others and avoiding them, which, in turn, leads to negative emotions, such as anxiety and irritability. The mood-congruent theory proposes that individuals tend to obtain information that is consistent with their own mood and show corresponding behaviors (Bower et al., 1978; Wei et al., 2019). Therefore, when employees experience negative emotions, they will make negative comments on their colleagues and organization, thereby reduce their willingness to help others and make more contributions to the organization (Borman et al., 2001), such as knowledge sharing (Trougakos et al., 2015; Wei et al., 2019). In conclusion, the self-maintenance tendency of a prevention focus does not fit the other-oriented tendency of prosocial motivation. When the two types of potential consciousness conflict, prevention focus weakens the effect of prosocial motivation on knowledge-sharing behavior to some extent. Based on the above analysis, the following hypothesis was proposed:

**Hypothesis 4**. Prevention focus negatively moderates the relationship between prosocial motivation and knowledge sharing. Compared with employees with a high prevention focus, prosocial employees with a low prevention focus are more likely to show knowledge sharing.

**Figure 1:**
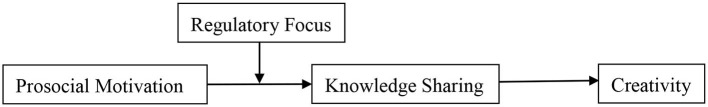
Research framework.

Based on the above theoretical derivation, the theoretical model for this study is shown in Figure 1. In summary, this study combined the motivated information processing model, componential theory of creativity, and regulatory focus theory to construct a moderated mediation model to analyze the effect of prosocial motivation on creativity (Figure 1). First, the current study explored the influence of prosocial motivation on the creativity of employees in the context of controlling for intrinsic motivation to analyze the unique role of prosocial motivation. Second, the study focused on knowledge sharing as a mediating variable to explore the mechanism by which prosocial motivation affects creativity. In this regard, we also constructed a main logical analysis framework based on the “motivation-behavior result.” Finally, based on the perspective of regulatory fitting and combined with regulatory focus theory, the study analyzed the moderating role of regulatory focus in the relationship between prosocial motivation and knowledge sharing. We also clarified the boundary factors of the prosocial motivation of employees that affect knowledge sharing.

## Methods

### Data and Sample

The data for this study were obtained from employees in China who were mainly working in the real estate, technology, manufacturing, trade, construction, and financial industries. The samples were selected mainly through random sampling. First, we contacted the enterprise managers through an enterprise service center. Then, through face-to-face or telephonic interviews with the enterprise managers, the research purpose and the objective of the study were explained in detail. Through the same methods, we also promised that the data filled in by employees would only be used for research and not for other commercial purposes. Finally, after obtaining the consent of the managers, according to the list of employees provided by each enterprise, we adopted a random sampling method to select employees for the questionnaire survey. At the same time, to expand the samples, we used convenience sampling as a supplement and collected data mainly from MBA (Master of Business Administration) students who were employees in the companies and acquaintances working in the enterprises. The questionnaire survey was conducted from September 2020 to January 2021.

To improve the recovery rate and quality of the questionnaire responses, the survey was conducted anonymously. Before administering the questionnaire, the employees were explained that the anonymity and confidentiality of the survey and results would only be used for academic purposes and had nothing to do with any assessment. At the same time, to avoid possible psychological implications caused by variable names, specific variable names were hidden in the questionnaire. In addition, we also requested the employees to answer the questionnaire carefully according to the actual situation to improve the quality of the responses. Regarding the sample size, it was required that the sample size of the model should be 5–10 times of the parameters to be estimated (Everitt, 1975). The model in this study included 38 observation variables; thus, 360 questionnaires were sent out, while 358 questionnaires were returned. To improve the accuracy and rigor of the survey, 38 invalid questionnaires were removed, leaving 320 valid questionnaires, with an effective recovery rate of 88.9%. The survey included 48.75% male and 51.25% female respondents. Most of the participants were below 30 years of age (90%) and 75% of them had a bachelor's degree or above. Their work experience was mainly within 5 years (80.63%). The overall distribution of jobs was even, with relatively many technical research and development positions (40.62%). Most of the employees were also labor staff (59.69%).

### Measures

The scales used in this study were adapted from scales that have been used by many scholars and validated in the Chinese context. Suitable modifications were made to fit the context of the current study. To improve the reliability of the measurement tools, the original English scale was translated into Chinese by a professional English language translator and a doctor studying abroad in the field of creativity research through a standard “translation-back translation” procedure before the formal distribution of the questionnaires. We then invited professors in the field of creativity research for repeated discussions about the questionnaire content and measurement tools. We also revised and adjusted the language expression of the questionnaire several times to ensure the rationality, standardization, and comprehensibility of the questions. Next, we interviewed five employees on their opinions about the readability and clarity of the language expression of the questionnaire to ensure the respondents fully understood the survey content. Finally, we developed the final questionnaire for the study. It should be noted that since the employees have a clearer perception of their own behaviors and motivations than their supervisors, and that the evaluations of supervisors or colleagues may be influenced by personal preferences or deluded by superficial behaviors, this study adopted an employee self-evaluation method. All items were scored on a five-point Likert scale, ranging from 1 (strongly disagree) to 5 (strongly agree).

Prosocial motivation was measured, using a five-item scale adapted from Grant and Sumanth (2009), which includes items such as “I get energized by working on tasks that have the potential to benefit others” and “I prefer to work on tasks that allow me to have a positive impact on others.” We measured knowledge sharing using the scale produced by the Chinese scholars Cao and Xiang (2014), including four items (for example, “I often share my work experience with colleagues or collaborators”), which were more suitable for the Chinese context. Regulatory focus was measured based on the 12-item scale of Mao (2016) adapted from Johnson and Chang (2008), in which promotion focus (for example, “I see work as a way to realize my wishes and ambitions”) and prevention focus (for example, “I am concerned about my failure experience at work”) were assessed using six items for each. The creativity of employees was also assessed based on the seven-item scale of Chen (2006), which was adapted from Tierney et al. (1999). One item was “I often come up with some creative ideas at work.”

Previous studies have shown that intrinsic motivation has an impact on employee creativity (Grant and Berry, 2011; Li and Bai, 2015a,b). Therefore, intrinsic motivation was used as the control variable in this study. It was measured using the scale developed by Grant (2008), including four items (for example, “I enjoy my job”). In addition, individual characteristics may affect employee creativity in the workplace. Therefore, the gender, age, education, and tenure of the employees were also considered as control variables (Wang, 2018; Ma and Yan, 2020).

### Reliability and Validity Analysis

Different forms of instrument validity and reliability were assessed for this study. Based on the criteria suggested by Fornell and Larcker (1981), Cronbach's α and composite reliability (CR) should be >0.7 and the average variance extracted (AVE) >0.5. Table 1 shows that Cronbach's α and CR for all constructs were >0.7 and the AVE of each construct was larger than 0.5, which are all higher than the values recommended by Fornell and Larcker (1981). Thus, the construct reliability and convergent validity of our instrument were acceptable.

**Table 1:** Reliability and convergent validity.

**Variable**	**Cronbach's α**	**CR**	**AVE**
Prosocial motivation	0.867	0.870	0.573
Creativity	0.890	0.891	0.539
Knowledge sharing	0.794	0.804	0.514
Promotion focus	0.857	0.858	0.502
Prevention focus	0.866	0.867	0.521

To investigate the discriminative validity of the discussed variables, this study used AMOS 24.0 to conduct a confirmatory factor analysis on the measured data and compare the fitting degrees of various nested models. Table 2 indicates that the five-factor model exhibited an optimal fit, demonstrating desirable discriminant validity among the constructs in this study.

**Table 2:** Confirmatory factor analysis results of the competition model.

**Model**	**χ2/df**	**GFI**	**CFI**	**TLI**	**RMSEA**	**SRMR**
Single-factor^a^	6.417	0.565	0.563	0.528	0.130	0.124
Two-factor^b^	5.237	0.624	0.659	0.631	0.115	0.113
Three-factor^c^	4.096	0.690	0.752	0.730	0.099	0.102
Four-factor^d^	3.513	0.715	0.801	0.781	0.089	0.095
Five-factor^e^	1.845	0.877	0.934	0.926	0.051	0.053
CMV model	1.800	0.881	0.937	0.930	0.050	0.052

a *Prosocial motivation + knowledge sharing + promotion focus + prevention focus + creativity*.

b *Prosocial motivation + knowledge sharing + promotion focus + prevention focus, creativity*.

c *Prosocial motivation, knowledge sharing + promotion focus + prevention focus, creativity*.

d *Prosocial motivation, knowledge sharing, promotion focus + prevention focus, creativity*.

e *Prosocial motivation, knowledge sharing, promotion focus, prevention focus, creativity*.

*CMV represents the common method variance*.

### Common Method Variance

Since each employee knows themselves best, all data were based on the self-reports of the employees. However, we took some measures to reduce the common method bias. On the one hand, the employees were asked to answer the questions according to the actual situation as their answers were only for academic purposes before administering the questionnaire. On the other hand, all variable names were hidden to prevent the employees from guessing what was being investigated. After questionnaire collection and selection, several statistical methods were used to test the potential common method bias, which normally exists in self-reported data (Podsakoff et al., 2003). First, we used a single-common-method-factor approach. The results showed that, after the common method deviation latent variable (CMV) was added to the theoretical model, the variation ranges of the fitting indices, such as RMSEA, CFI, and GFI of the model, were all below 0.006 (as shown in Table 2), indicating that potential common method bias was unlikely to occur in this study. Second, we examined the correlation coefficient between variables. If the correlation coefficient is higher than 0.9, it means that the common method deviation problem is serious (Liu et al., 2021). The analysis results of this study showed that the highest correlation coefficient between the main variables was 0.553, which was lower than the critical value of 0.9. In addition, we also conducted collinearity diagnostics and found that the VIF value of all the variables was not more than 2.268, which is much lower than the critical value of 10 (Zhang et al., 2020). In summary, there was no serious common method bias in the research data, and no serious collinearity occurred among any of the constructs.

### Analytic Strategy

In this study, SPSS26.0, which was used to analyze the reliability and bivariate correlations between all factors, and AMOS 24.0 were used to analyze the validity of the data. We tested our model and hypotheses using the computer program PROCESS, which has been accepted and applied by many scholars because it is more scientific, effective, and convenient. This program provides standard tests and bootstrap confidence intervals (CIs), which were based on 5,000 samplings, for individual regression. Furthermore, if the lower and upper limits of the confidence interval in our study did not include zero, the corresponding effect was significant (Chen et al., 2013).

## Results

### Descriptive Statistics

Table 3 presents the means, standard deviations, and correlations among the variables. Prosocial motivation, employee creativity, regulatory focus, and knowledge sharing had high correlations. There was a significant positive correlation between prosocial motivation and employee creativity (β = 0.478, *p* < 0.01). Prosocial motivation was also positively correlated with knowledge sharing (β = 0.451, *p* < 0.01), while knowledge sharing was positively correlated with employee creativity (β = 0.553, *p* < 0.01), which provided preliminary evidence for the subsequent exploration of the relationships of the variables.

**Table 3:** Means, standard deviations (SD), and correlations.

**Variables**	**1**	**2**	**3**	**4**	**5**	**6**	**7**	**8**	**9**	**10**	**11**	**12**
Gender	1											
Age	−0.146^**^	1										
EDU	−0.009	−0.290^**^	1									
Tenure	−0.137^*^	0.681^**^	−0.468^**^	1								
Post	0.182^**^	−0.012	−0.172^**^	−0.027	1							
Position	−0.074	0.024	0.003	−0.046	0.217^**^	1						
IM	−0.080	0.095	−0.047	0.093	−0.012	0.047	1					
PSM	−0.040	0.134^*^	0.042	0.058	−0.050	−0.045	0.407^**^	1				
KS	−0.044	0.084	−0.078	0.100	−0.040	0.009	0.376^**^	0.451^**^	1			
PRO	0.023	0.069	0.037	0.017	−0.074	0.016	0.404^**^	0.500^**^	0.526^**^	1		
PRE	−0.066	−0.034	−0.017	−0.070	0.128^*^	0.029	0.139^*^	0.106	0.281^**^	0.339^**^	1	
CR	−0.161^**^	0.078	0.004	−0.011	−0.132^*^	0.060	0.472^**^	0.478^**^	0.553^**^	0.515^**^	0.161^*^	1
Mean	1.51	1.89	2.92	1.97	3.89	1.92	3.524	4.147	3.942	4.241	3.671	3.698
SD	0.501	0.751	0.817	0.998	2.083	1.416	0.994	0.734	0.678	0.629	0.764	0.751

* *p < 0.05*,

** *p < 0.01*.

*IM, intrinsic motivation; PSM, prosocial motivation; CR, creativity; KS, knowledge sharing; PRO, promotion focus; PRE, prevention focus; EDU, education*.

### Main Effect and Mediating Effect

First, Model 4 in the PROCESS program (Model 4 is a simple mediation model) was used to test the main effects and mediating effects of knowledge sharing after controlling for intrinsic motivation, gender, age, etc. As shown in Tables 4, 5, the results showed that Hypothesis 1 (H1) and Hypothesis 2 (H2) were supported. Specifically, from M2 in Table 4, prosocial motivation significantly and positively affected creativity [effect = 0.350, CI (95%) = (0.249, 0.451)]. Thus, H1 was verified. From M1 in Table 4, the regression coefficient of prosocial motivation on knowledge sharing was significant [effect = 0.339, CI (95%) = (0.241, 0.438)]. From M3 in Table 4, the regression coefficient of knowledge sharing on creativity was also significant (effect = 0.404, CI (95%) = (0.299, 0.510)]. From Table 5, the indirect effect of prosocial motivation on creativity was 0.137, with the bootstrap CI for this coefficient being [CI (95%) = (0.079, 0.214)], while the direct effect of prosocial motivation on creativity after controlling for the mediator variable was also significant (effect = 0.213), with its bootstrap confidence interval being [CI (95%) = (0.113, 0.313)]. Thus, H2 was supported, indicating that knowledge sharing plays a mediating role between prosocial motivation and creativity.

**Table 4:** Main effects and mediation effects.

**Variables**	**Outcome, knowledge sharing**			**Outcome, creativity**					
	**M1**			**M2**			**M3**		
	**Effect value**	**se**	**95% CI**	**Effect value**	**se**	**95% CI**	**Effect value**	**se**	**95% CI**
Constant	2.232^***^	0.305	[1.632, 2.832]	2.017^***^	0.314	[1.400, 2.634]	1.115^***^	0.313	[0.499, 1.731]
Gender	−0.004	0.069	[−0.139, 0.131]	−0.159^*^	0.071	[−0.298, −0.020]	−0.157^*^	0.065	[−0.285, −0.029]
Age	−0.042	0.061	[−0.162, 0.079]	0.068	0.063	[−0.055, 0.192]	0.085	0.058	[−0.029, 0.199]
Education	−0.064	0.047	[−0.157, 0.028]	−0.065	0.049	[−0.161, 0.030]	−0.039	0.045	[−0.127, 0.049]
Tenure	0.036	0.050	[−0.062, 0.134]	−0.117^*^	0.051	[−0.218, −0.017]	−0.132^**^	0.047	[−0.225, −0.039]
Post	−0.012	0.017	[−0.046, 0.021]	−0.044^*^	0.018	[−0.079, −0.010]	−0.039^*^	0.016	[−0.071, −0.008]
Position	0.013	0.024	[−0.035, 0.061]	0.037	0.025	[−0.012, 0.087]	0.032	0.023	[−0.013, 0.077]
IM	0.150^***^	0.037	[0.078, 0.222]	0.245^***^	0.038	[0.170, 0.319]	0.018^***^	0.036	[0.114, 0.255]
PSM	0.339^***^	0.050	[0.241, 0.438]	0.350^***^	0.052	[0.249, 0.451]	0.213^***^	0.051	[0.113, 0.313]
KS							0.404^***^	0.054	[0.299, 0.510]
F		13.508^***^			21.926^***^			29.262^***^	
*R* ^2^		0.258			0.361			0.459	

* *p < 0.05*,

** *p < 0.01*,

*** *p < 0.001*.

*PSM, prosocial motivation; CR, creativity; KS, knowledge sharing; PRO, promotion focus; PRE, prevention focus*.

**Table 5:** Results of the hypothesized relationships between prosocial motivation, knowledge sharing, and creativity.

	**EFFECT**	**SE**	**LLCI**	**ULCI**
Total effect	0.350	0.052	0.249	0.451
Direct effect	0.213	0.051	0.113	0.313
Indirect effect	0.137	0.034	0.079	0.214

### Moderating Effect

We used Model 7 in the PROCESS program to test the moderating effects of regulatory focus after controlling for intrinsic motivation, gender, age, etc. First, we checked the moderating effect of promotion focus. From M1 in Table 6, we found that the interaction between prosocial motivation and promotion focus was significant [effect = −0.129, CI (95%) = (−0.243, −0.014)], indicating that promotion focus had a negative moderating effect on the relationship between prosocial motivation and knowledge sharing, which is the opposite of Hypothesis 3 (H3). As shown in Table 7, when the level of promotion focus was low, the effect of prosocial motivation on knowledge sharing was significant [effect = 0.111, CI (95%) = (0.049, 0.191)]. However, when the promotion focus level was high, the effect of prosocial motivation on knowledge sharing was not significant [effect = 0.045, CI (95%) = (−0.016, 0.120)], which also indicated that promotion focus had a negative moderating effect on the relationship between prosocial motivation and knowledge sharing.

**Table 6:** Results of the hypothesized relationships between prosocial motivation, knowledge sharing, promotion focus, prevention focus, and creativity.

	**Outcome, knowledge sharing**						**Outcome, creativity**		
**Variables**	**M1**			**M2**			**M3**		
	**Effect value**	**se**	**95% CI**	**Effect value**	**se**	**95% CI**	**Effect value**	**se**	**95% CI**
Prosocial motivation	0.193^***^	0.051	[0.093, 0.293]	0.301^***^	0.049	[0.205, 0.397]	0.213^***^	0.051	[0.113, 0.313]
Knowledge sharing							0.404^***^	0.054	[0.299, 0.510]
Promotion focus	0.353^***^	0.062	[0.230, 0.476]						
PSM × PRO	−0.129^*^	0.058	[−0.243, −0.014]						
Prevention focus				0.211^***^	0.043	[0.127, 0.296]			
PSM × PRE				−0.132^**^	0.050	[−0.231, −0.033]			
R	0.602			0.568			0.678		
*R* ^2^	0.362			0.323			0.459		
F	17.566^***^			14.725^***^			29.262^***^		

* *p < 0.05*,

** *p < 0.01*,

*** *p < 0.001*.

*PSM, prosocial motivation; CR, creativity; KS, knowledge sharing; PRO, promotion focus; PRE, prevention focus*.

**Table 7:** Conditional effect of prosocial motivation on knowledge sharing based on regulatory focus.

	**Moderator: promotion focus**	**EFFECT**	**SE**	**LLCI**	**ULCI**
	−1SD (−0.65)	0.111	0.036	0.049	0.191
	M (0)	0.078	0.029	0.028	0.143
**Outcome: knowledge sharing**	+1SD (+0.65)	0.045	0.034	−0.016	0.120
	**Moderator: prevention focus**	**EFFECT**	**SE**	**LLCI**	**ULCI**
	−1SD (−0.78)	0.162	0.044	0.088	0.259
	M (0)	0.122	0.031	0.069	0.194
	+1SD (+0.78)	0.081	0.037	0.020	0.168

We then examined the moderating effect of prevention focus. From M2 in Table 6, the interaction between prosocial motivation and prevention focus was significant [effect = −0.132, CI (95%) = (−0.231, −0.033)], indicating that prevention focus negatively moderated the relationship between prosocial motivation and knowledge sharing. Thus, Hypothesis 4 (H4) was verified. Table 7 also shows that, when the level of prevention focus was low, the effect of prosocial motivation on knowledge sharing was significant [effect = 0.162, CI (95%) = (0.088, 0.259)]. However, as the level increased, the effect of prosocial motivation on knowledge sharing decreased (from 0.162 to 0.081). The difference in the conditional effects of prosocial motivation on knowledge sharing also indicated that prevention focus had a negative moderating effect.

**Figure 2:**
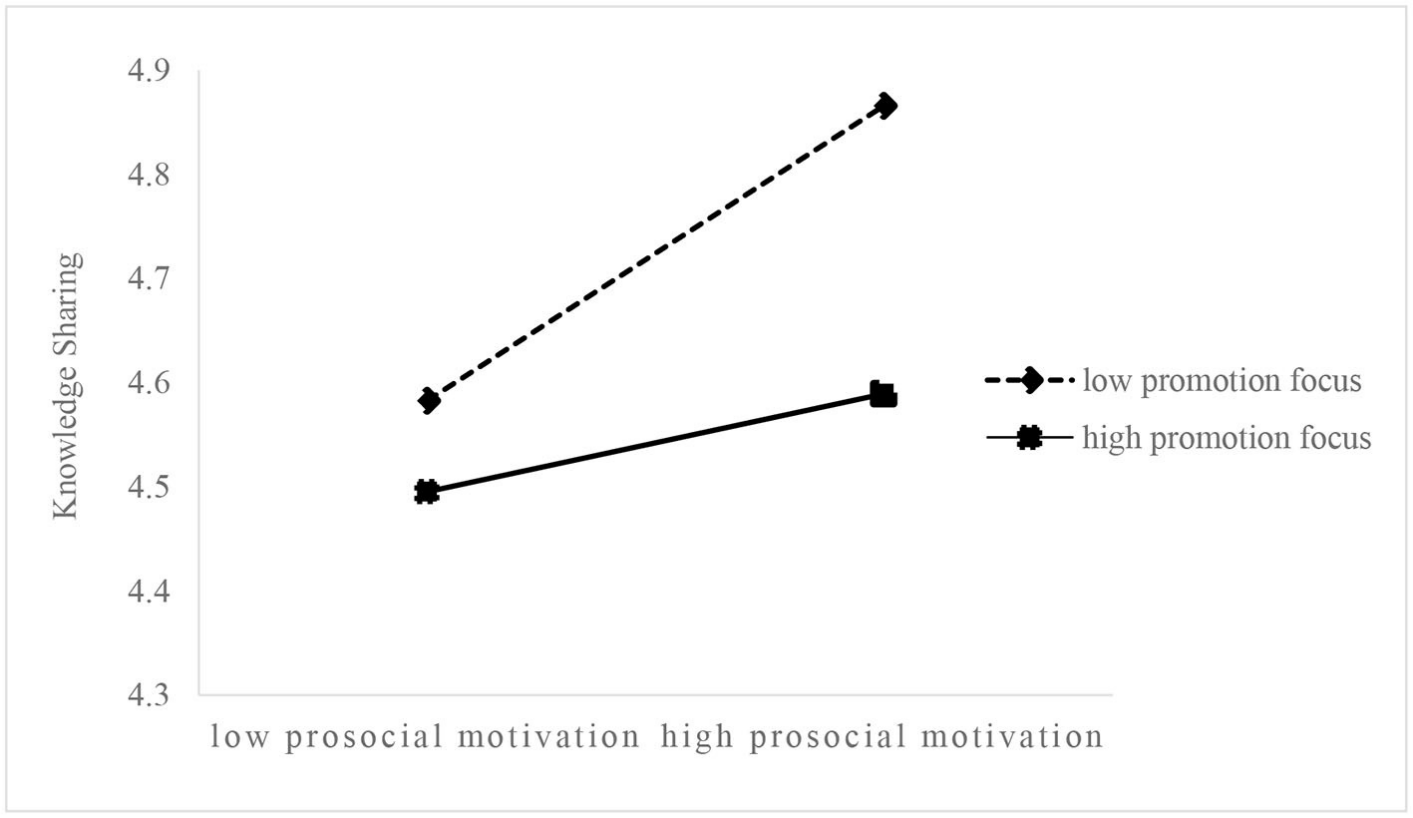
Moderating effect of promotion focus on the relationship between prosocial motivation and knowledge sharing.

**Figure 3:**
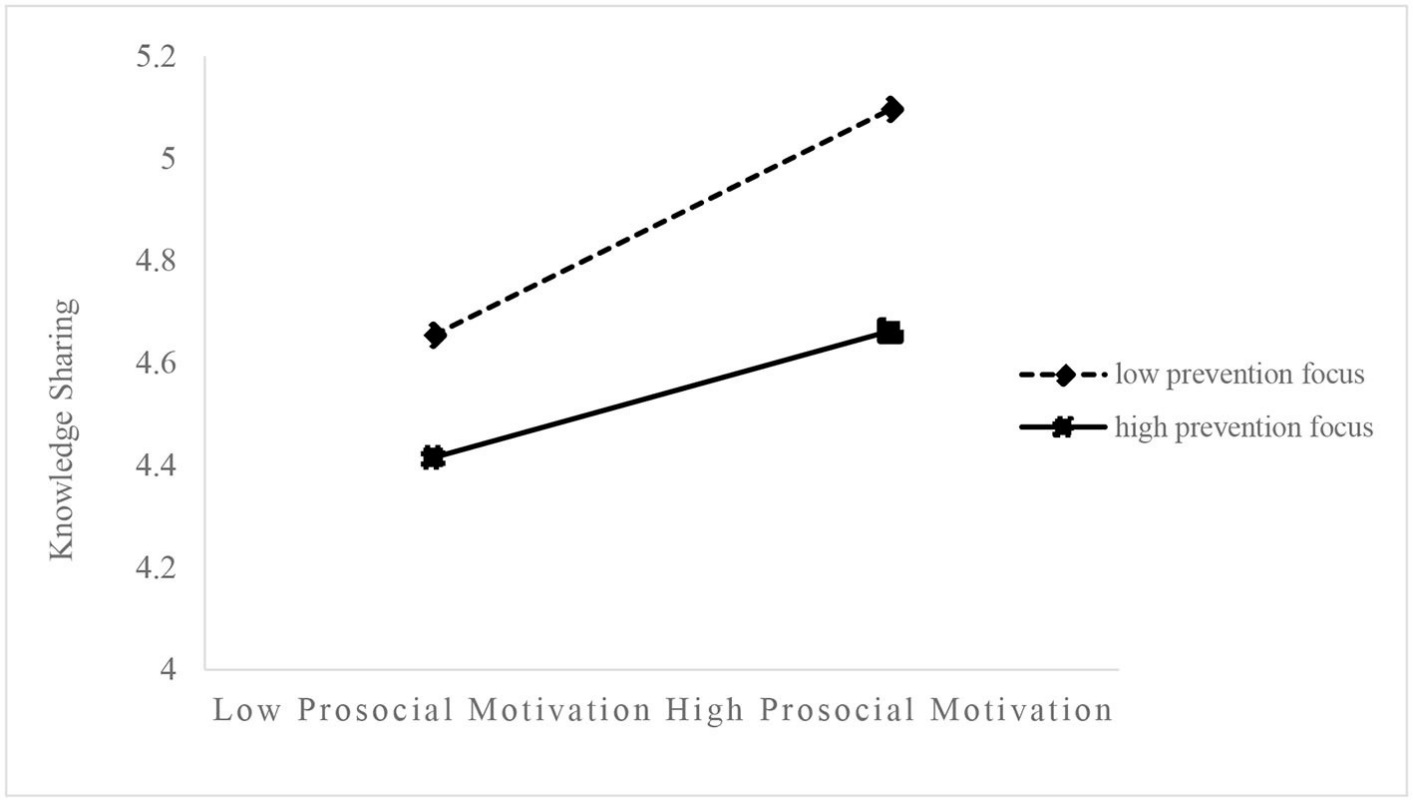
Moderating effect of prevention focus on the relationship between prosocial motivation and knowledge sharing.

To clearly explain the moderating effect of regulatory focus on the relationship between prosocial motivation and knowledge sharing, we plotted simple slopes to show the relationship between prosocial motivation and knowledge sharing at high (mean + SD) and low (mean – SD) levels of regulatory focus (Figures 2, 3). Figure 2 illustrates that, compared with the employees with a high promotion focus, the regression slope between prosocial motivation and knowledge sharing is greater for the employees with low promotion focus. Similarly, Figure 3 shows that, compared with the employees with a high prevention focus, the regression slope between prosocial motivation and knowledge sharing is greater for the employees with a low prevention focus.

## Discussion and Implications

### Discussion

Based on the motivated information processing model, componential theory of creativity, and regulatory focus theory, this study attempted to explore the relationship between prosocial motivation and employee creativity and its mechanisms. Through an empirical analysis of the data of 320 employees, we tested the relationship between prosocial motivation and creativity, the mediating role of knowledge sharing in the aforementioned relationship, and the moderating role of regulatory focus on the relationship between prosocial motivation and knowledge sharing under the condition of controlling for intrinsic motivation. Several key findings can be derived from the results of this study.

First, after controlling for intrinsic motivation, prosocial motivation was positively correlated with creativity, a result that supported H1. This conclusion deepened the research conclusion of Li and Bai (2015a,b) that prosocial motivation has a significant impact on creativity when exploring the relationship between intrinsic motivation and creativity. This finding also verified the conclusion of Liu et al. (2016) that prosocial motivation has a unique effect on creativity through meta-analysis. This shows that employees with prosocial motivation are willing to engage in work that is beneficial to others, participate in projects that can bring benefits to others, and can get sufficient energy from these helping jobs. Consequently, to better create well-being and benefits for others in the future, they often come up with creative ideas or try new ideas, procedures, or methods in their work to effectively promote the interests of others. Therefore, from an empirical perspective, this conclusion further demonstrated that prosocial motivation is an important predictor of creativity after controlling for intrinsic motivation.

Second, knowledge sharing played a partially mediating role in the relationship between prosocial motivation and creativity, with the direction of this relationship being consistent with our H2. This conclusion clarified the previous fuzzy conclusion that prosocial value orientation may positively affect knowledge sharing (Jadin et al., 2013), further confirmed that prosocial motivation has a positive effect on knowledge sharing, and verified once more that knowledge sharing is an important path for the formation of employee creativity (Zhu and Chen, 2013; Bhatti et al., 2020). This shows that prosocial motivation encourages employees to actively pay attention to the interests and needs of others and seek opportunities to do their best to help others. With this, employees become willing to share new information and accumulated experience at work when their collaborators or colleagues need them and often express their suggestions and opinions in group discussions. Furthermore, in the process of sharing, these employees become more aware of the practical needs of their colleagues, which, in turn, leads to their creative solutions for the difficulties of these colleagues. In addition, after these employees express their opinions, the suggestions of others may make up for what may have been lacking in previous thinking, which provides a reference for them to come up with other creative ideas later on.

Third, promotion focus negatively moderated the relationship between prosocial motivation and knowledge sharing, with H3 not being supported. This suggests that the positive effect of prosocial motivation on knowledge sharing weakens when employees excessively pursue success and consider the benefits of work, which is contrary to our research hypothesis. This may be because employees with a high promotion focus mainly meet the needs of self-growth at work, have a strong ideal self, are very sensitive to rewards, and regard work success as the most important achievement in life (Higgins, 1998). However, prosocial motivation reflects care for other of individuals. Specifically, prosocial individuals tend to move beyond the limitations of their own perspectives, expect their work to bring welfare to others, are willing to participate in work beneficial to others, and try their best to make sure that their work contributes to the well-being of others (Grant, 2008). The high attention of employees with high promotion focus on self-interest does not match the prosocial motivation of interest appeal aimed at the welfare of others. Therefore, although employees with prosocial motivation want to share knowledge or help others, they are likely to reduce the degree of knowledge sharing or even hide knowledge based on self-interest and their pursuit of success. Thus, H3 was not supported.

Finally, prevention focus had a negative moderating effect on the relationship between prosocial motivation and knowledge sharing, with this result supporting H4. Individuals with high prevention focus are always worried about negative results at work (e.g., losing their jobs) and often feel anxious, irritable, and other negative emotions, consequently making them more likely to adopt avoidance strategies at work (Higgins, 1998). This does not match with prosocial motivation, which is characterized by the willingness to help others and the desire to create well-being for others. Therefore, when the prevention focus of an individual is high, prosocial motivation will conflict with the behavioral tendency of prevention focus. This leads to prevention focus weakening the prosocial motivation to promote knowledge sharing behavior to some extent. It is worth noting, however, that Table 6 shows a positive relationship between prevention focus and knowledge sharing. The possible reasons for this are as follows. First, individuals with a prevention focus often set challenging goals at work, pay more attention to existing problems, and try to avoid those problems (Li and Zhong, 2020). Studies have found that, to reduce the risk of failure in the market environment and prevent the occurrence of mistakes, they will constantly monitor, summarize, and reflect on their internal and external environments, workflows, and methods, form their own experience, and communicate with others (He and Liu, 2021). Koopmann et al. (2019) also found that prevention focus was positively correlated with voice behavior. In fact, individuals with a prevention focus will point out and stop unrealistic suggestions for the interests of their enterprise and show extra-role behaviors (e.g., prohibitive voice behavior and knowledge sharing) to maximize the effect of timely loss stopping (Lin and Johnson, 2015; MacMillan et al., 2019). Second, individuals with a prevention focus are more sensitive to negative information, such as being scolded by leaders and ostracized by colleagues, which they try to avoid (Lanaj et al., 2012; Hamstra et al., 2014). Such a perception tendency also makes them more concerned about their own reputations and avoids the formation of bad images (Pfattheicher, 2015). Therefore, if prevention-focused employees do not share knowledge, they are prone to negative perceptions (Cho, 2006), such as being labeled as stingy and even losing the trust of others (Connelly et al., 2012; Guo et al., 2020). In response, they will exhibit more knowledge-sharing behaviors to avoid damaging their images and reputations. It is worth noting that, while the results of this study did show that prevention focus was positively correlated with knowledge sharing, some scholars have found that the two are not correlated (Li et al., 2014a). This also provides a new direction for future research to explore the relationship between the two.

### Theoretical Contributions

Our study on the mechanisms in the relationship between prosocial motivation and creativity has some theoretical contributions. First, we validated the unique relationship between prosocial motivation and creativity under the condition of controlling for intrinsic motivation. On the one hand, this result explained the inconsistency between the research results on the relationship between prosocial motivation and creativity and supported the discussion of scholars on the positive relationship between the two (De Dreu et al., 2011; Grant and Berg, 2011; Grant and Berry, 2011; Li and Bai, 2015a,b). On the other hand, unlike the previous belief that prosocial motivation can enhance the relationship between intrinsic motivation and creativity (Grant and Berry, 2011; Li and Bai, 2015a), we controlled for intrinsic motivation and concluded that prosocial motivation and creativity are positively correlated, which enhanced our understanding of prosocial motivation. In addition, individuals living in the Chinese traditional culture are influenced by collectivism, which emphasizes the spirit of “helping others to be happy.” With such social norms, individuals attach great importance to prosocial behavior (Li et al., 2012) and are more susceptible to prosocial motivation. Furthermore, this study examined the relationship between prosocial motivation and creativity to verify the motivational information processing theory in the Chinese context, thus providing a new empirical basis for the applicability of the theory to Eastern cultures.

Second, this study introduced knowledge sharing into the theoretical framework and discussed the mechanism of prosocial motivation in employee creativity, which compensates for the lack of attention given to the mediation mechanism in the existing literature. Scholars have proposed that, to better understand the process of prosocial motivation-stimulating creativity, more attention should be paid to the unique mechanism of prosocial motivation (Liu et al., 2016); however, previous studies have rarely discussed this in depth. In this regard, this study extensively analyzed the mediating role of knowledge sharing in the relationship between prosocial motivation and creativity, thus making up for the lack of existing research and expanding relevant theories. Specifically, knowledge sharing is a risky and autonomous behavior that often requires strong internal motivation (Bock et al., 2005). Additionally, scholars have called for the imperative application of motivational theories from psychology to knowledge sharing (Wang and Noe, 2010). Therefore, we applied the motivated information processing model to find out the impact of employee prosocial motivation on knowledge sharing. Our study not only enriched previous studies on the relationship between altruism and knowledge sharing (Wang and Hou, 2015) but also promoted the application of this theory in the Chinese context and opened a new perspective for subsequent research. More importantly, based on the componential theory of creativity, this study explored the importance of knowledge sharing in enhancing employee creativity and verified the conclusions of previous studies on the relationship between knowledge sharing and creativity (Zhu and Chen, 2013; Zhang et al., 2016). As a result, a complete transmission mechanism of the “motivation-behavior result” has been formed (Liu and Chi, 2019), indicating that knowledge sharing is one of the important paths for the formation of employee creativity (Zhu and Chen, 2013; Bhatti et al., 2020) and consequently revealing the “black box” between prosocial motivation and creativity.

Finally, this study also introduced the regulatory focus theory to explore the boundary conditions between the prosocial motivation and knowledge sharing of employees, thus enriching the research on the relationship between regulatory focus and knowledge sharing. When analyzing the relationship between regulatory focus and knowledge sharing, existing literature focused on the direct role of regulatory focus on knowledge sharing (Li et al., 2014b) and examined the moderating role of regulatory focus between organizational elements and knowledge sharing (Ju et al., 2019). However, little attention has been paid to the moderating role of regulatory focus in the relationship between individual motivational factors and knowledge sharing. Therefore, this study investigated the moderating effect of regulatory focus on the relationship between prosocial motivation and knowledge sharing, which can compensate for the deficiencies in existing literature to a certain extent. At the same time, this study responded to the call of Brockner and Higgins (2001) to apply the regulatory focus theory to organizational research. In connection with this, from the perspective of regulatory fitting, this study introduced the employee regulatory focus as a moderating variable, which more comprehensively reflects the complex process of prosocial motivation affecting employee knowledge sharing. As a result, it was confirmed that, because of the mismatch between promotion and prevention focus and the interest demands of prosocial motivation, both promotion focus and prevention focus can weaken the influence of prosocial motivation on knowledge sharing when employees are faced with the difficult choice of whether to share the knowledge they own. In conclusion, this study not only deepened the research on the influencing factors of knowledge sharing, but also expanded the theory of regulatory focus in organizational behavior to a certain extent.

### Managerial Implications

Our research findings have several important managerial implications. First, our study showed that prosocial motivation increases the creativity of employees. Given the fact that behaviors that consider the interests of others are encouraged in China (Hofstede, 2001), we should pay attention to the stimulation of the prosocial motivation of employees not only to improve their creativity, but also to promote the harmonious development of society. Concretely, it is necessary to properly conduct talent selections and establish reasonable talent standards in the early stages of hiring. For instance, when the personnel department recruits new employees, in addition to emphasizing the necessary work skills, they should also pay attention to the selection of employees with prosocial motivation and the ability to think from the perspectives of others. If necessary, the traits of employees should be tested using certain evaluation forms. Specifically, in employee training, managers should pay attention to stimulating and shaping prosocial values gradually, guiding employees to develop the habit of perspective taking, and laying a good psychological and behavioral foundation for releasing creativity. In the actual production, design, service, and other processes during employment, managers should try to let employees think from the perspective of others (e.g., customers, colleagues, and organizations) (Grant and Berry, 2011) and understand the characteristics of their service objects and the meaning of their work to help others (Grant, 2008). These can enhance the prosocial motivation of employees, thereby promoting employee creativity and generating better ideas and solutions.

Second, this study strengthens the advocacy of employee knowledge-sharing behavior by revealing that knowledge sharing is an important mechanism through which prosocial motivation affects employee creativity and further clarifying the role of knowledge sharing in organizations. Promoting employee knowledge sharing has become an important topic of practical concern. Specifically, organizations can encourage employees to share knowledge from two aspects: willingness and the path of knowledge sharing. On the one hand, knowledge sharing is not only an active behavior, but also an organizational citizenship behavior or a prosocial behavior outside of the responsibilities of employees (Bolino and Grant, 2016; Peng et al., 2019). Consequently, making such a behavior “voluntary” rather than “coercive” is the most significant feature (Zhao, 2013). Therefore, the management strategy of an organization for employee knowledge sharing should be encouraged rather than mandatory. In this regard, companies can develop and use incentives to promote employee knowledge sharing. The coordinated implementation of material and spiritual rewards can effectively stimulate knowledge sharing among employees. At the same time, organizations can create a shared or diversified organizational atmosphere and culture to reduce group prejudice behavior, increase emotional trust among members, and guide employees to participate in knowledge sharing, thus improving employee creativity. On the other hand, organizations should also improve knowledge-sharing channels and create conditions for knowledge sharing, which can be done by designing appropriate organizational structures and strengthening cognitive learning through the optimization of technical systems. In general, motivating employee knowledge sharing is a systematic process that needs to be promoted from multiple perspectives. Based on an in-depth study of the micro mechanism of knowledge sharing, an organization can systematically construct a knowledge governance system from the organizational structure, working relationships, cognitive learning, incentive mechanism, organizational culture, and other aspects so as to form a conducive environment where employees have both the willingness and the opportunity to share, thereby enhancing the level of organizational creativity.

Finally, managers can focus on exerting an influence on the individual behavioral tendencies of employees. This research showed that both promotion and prevention focus play a negative moderating role in the relationship between prosocial motivation and knowledge sharing. Specifically, employees with high promotion focus place too much emphasis on their own success, are too utilitarian, and take knowledge and experience as their own in order to achieve their own goals regardless of the needs of others, with the behaviors not being conducive to knowledge sharing and creativity. Meanwhile, employees with high prevention focus fear failure and are timid, worrying that knowledge sharing will threaten existing interests. Therefore, organizations can avoid hiring candidates with high prevention focus or promotion focus, using a scale to measure the focus orientation of candidates when necessary. Furthermore, individuals with high regulatory focus place too much emphasis on their own interests, thus forming strong self-interest-centered principles. In this regard, organizations can also guide the regulatory focus of employees through reinforcement and training (Johnson et al., 2015; Song et al., 2019); for example, through publicity and education, the organization of activities, and increasing team performance evaluations to make employees realize that knowledge sharing is not only a contribution, but also a mutually beneficial and win-win process, with collective success being conducive to the individual development of employees. At the same time, attention should be paid to avoiding polarization during training, which also fits the Chinese traditional culture of the doctrine that states that everything has a degree, and that if you go too far, you cannot get enough. In addition, a team leader can show not only the behavior that focuses on collective interests, but also avoid selfish behaviors that sacrifice the interests of their subordinates during work. Such a role model can reduce the guard and utilitarianism of members so that the regulatory focus of employees can be maintained at a proper level.

### Limitations and Future Research Directions

In this study, we controlled for intrinsic motivation and confirmed the relationship and mechanism between prosocial motivation and creativity. The above results are generally meaningful, but there are also some shortcomings. (1) All the cross-sectional data of the variables were collected in the same period, which limited the discussion of causality between the variables. In the future, longitudinal research should be considered as much as possible, and the different data of the variables should be obtained at different time points. (2) The research method was a single-questionnaire survey method. By using the form of questionnaires, employee self-reporting was, inevitably, a social applicability problem. In the future, we should combine various research methods as much as possible, such as a field experiment, and combine self-reporting with the evaluation. (3) In this study, promotion focus played a negative moderating role, which was contrary to the research hypothesis. Although this seems to relate to the unique situation in China, it needs to be verified further in future research.

Finally, it should be pointed out that this study was only a preliminary discussion of the mechanisms of prosocial motivation and creativity. There is still a large research space on the mechanisms between the two. For example, in the context of complex motivation, it may be interesting to study how the prosocial motivation, intrinsic motivation, approach motivation, and avoidance motivation of employees together affect creativity. In addition, complex creative activities in real life are mostly group activities; thus, the effect of individual prosocial motivation on group creative performance can become a new direction of research. However, the research object of group creation activities involves two levels: the creative performance of a group and the individual creative performance in the group. In a follow-up study, when discussing the role of prosocial motivation in group creation, it is necessary to evaluate the creative performance of the group together with individual performance. Therefore, we need to explore more deeply the relevant theories of prosocial motivation.

## Data Availability Statement

The original contributions presented in the study are included in the article/supplementary material, further inquiries can be directed to the corresponding author/s.

## Author Contributions

XQP and XZT recruited the participants, collected the data, and prepared the draft. XPP performed the data analysis. XZT reviewed the data critically and gave important advice. All authors contributed to the article and approved the submitted version.

## Funding

This research was supported by the National Natural Science Foundation of China (Grant No. 71872023), Chongqing Research Innovation Project for Postgraduate Students (Grant No. CYS21385), and Tianjin Research Innovation Project for Postgraduate Students (Grant No. 2019YJSB088).

## Conflict of Interest

The authors declare that the research was conducted in the absence of any commercial or financial relationships that could be construed as a potential conflict of interest.

## Publisher's Note

All claims expressed in this article are solely those of the authors and do not necessarily represent those of their affiliated organizations, or those of the publisher, the editors and the reviewers. Any product that may be evaluated in this article, or claim that may be made by its manufacturer, is not guaranteed or endorsed by the publisher.
